# An improved 7K SNP array, the C7AIR, provides a wealth of validated SNP markers for rice breeding and genetics studies

**DOI:** 10.1371/journal.pone.0232479

**Published:** 2020-05-14

**Authors:** Karina Y. Morales, Namrata Singh, Francisco Agosto Perez, John Carlos Ignacio, Ranjita Thapa, Juan D. Arbelaez, Rodante E. Tabien, Adam Famoso, Diane R. Wang, Endang M. Septiningsih, Yuxin Shi, Tobias Kretzschmar, Susan R. McCouch, Michael J. Thomson

**Affiliations:** 1 Department of Soil and Crop Sciences, Texas A&M University, College Station, Texas, United States of America; 2 Plant Breeding and Genetics Section, School of Integrative Plant Sciences, Cornell University, Ithaca, New York, United States of America; 3 Rice Breeeding Platform, International Rice Research Institute, Los Baños, Philippines; 4 Texas A&M AgriLife Research Center, Beaumont, TX, United States of America; 5 Louisiana State University Ag Center, H. Rouse Caffey Rice Research Station, Rayne, LA, United States of America; ICAR-Indian Institute of Rice Research, INDIA

## Abstract

Single nucleotide polymorphisms (SNPs) are highly abundant, amendable to high-throughput genotyping, and useful for a number of breeding and genetics applications in crops. SNP frequencies vary depending on the species and populations under study, and therefore target SNPs need to be carefully selected to be informative for each application. While multiple SNP genotyping systems are available for rice (*Oryza sativa* L. and its relatives), they vary in their informativeness, cost, marker density, speed, flexibility, and data quality. In this study, we report the development and performance of the Cornell-IR LD Rice Array (C7AIR), a second-generation SNP array containing 7,098 markers that improves upon the previously released C6AIR. The C7AIR is designed to detect genome-wide polymorphisms within and between subpopulations of *O*. *sativa*, as well as *O*. *glaberrima*, *O*. *rufipogon* and *O*. *nivara*. The C7AIR combines top-performing SNPs from several previous rice arrays, including 4,007 SNPs from the C6AIR, 2,056 SNPs from the High Density Rice Array (HDRA), 910 SNPs from the 384-SNP GoldenGate sets, 189 SNPs from the 44K array selected to add information content for elite U.S. *tropical japonica* rice varieties, and 8 trait-specific SNPs. To demonstrate its utility, we carried out a genome-wide association analysis for plant height, employing the C7AIR across a diversity panel of 189 rice accessions and identified 20 QTLs contributing to plant height. The C7AIR SNP chip has so far been used for genotyping >10,000 rice samples. It successfully differentiates the five subpopulations of *Oryza sativa*, identifies introgressions from wild and exotic relatives, and is useful for quantitative trait loci (QTL) and association mapping in diverse materials. Moreover, data from the C7AIR provides valuable information that can be used to select informative and reliable SNP markers for conversion to lower-cost genotyping platforms for genomic selection and other downstream applications in breeding.

## Introduction

Single nucleotide polymorphisms (SNPs) occur at specific positions in the genome and are the most common form of genetic variation in eukaryotic organisms. They are the result of random mutations that occur every few hundred base pairs in most genomes [1]. While the majority of SNPs are found in the DNA between genes, they can also occur within genes and in regulatory regions where they may directly affect gene function and give rise to observable phenotypic variation.

In rice research communities, high-throughput sequencing and SNP genotyping assays over the last 20 years have generated large amounts of SNP data that can be used to calculate the frequency of SNP alleles in different populations, to track the inheritance of those alleles across generations, and to associate SNP variation with phenotypic variation [2]. This information provides the basis for developing high-value genotyping assays that target particular subsets of SNPs for a variety of downstream applications. Identifying the subsets of SNPs that are most informative, most reliable, and easiest to call is essential for designing optimal, cost-effective genotyping approaches for specific applications in genetics and breeding.

SNP assays for rice have been developed at different densities, with examples including the C6AIR [3], the RICE6K [4], the 44K-SNP chip [5], and the 700K-SNP High Density Rice Array [6]. Data generated using these SNP arrays are useful for genome wide association analysis, differentiating subpopulation groups, DNA fingerprinting, and genetic diversity analysis. Lower-density SNP arrays have also been widely used in rice genetics and breeding, including several 384-SNP bead sets used for QTL mapping, trait integration and confirmation of cultivar identity [7]. At this time, SNP arrays that provide genome-wide coverage and include markers that are informative both within and between the major subpopulations of rice are in high demand because they offer fast sample turn-around-time, and reliable, consistent, and easy to interpret data that can be readily databased. The SNP density required to meet these criteria in rice is ~6–7,000 markers, due to the significant differences in SNP distribution and frequency that characterize the deeply differentiated subpopulations of *O*. *sativa* [3].

The Cornell-IR LD Rice Array (C7AIR) improves upon the C6AIR [3] by removing poorly performing SNPs, maintaining the ability to differentiate between the five major subpopulations of *O*. *sativa*, and incorporating new SNPs that are informative for specific subgroups, particularly the US *tropical japonica* breeding community. The C7AIR targets a sweet spot between cost and informativeness for the global rice community. In contrast to *de novo* sequencing technologies that require a high level of computational and bioinformatics expertise, the C7AIR offers a high throughput genotyping assay with straightforward data analysis, and provides opportunities for immediate application in genebank management, genetic analysis, and applied plant breeding. Moreover, the C7AIR rice genotype data provides a valuable source from which to select validated SNP markers informative for specific germplasm groups for conversion to lower-cost assay systems for high-sample throughput applications.

## Materials and methods

### Plant materials

A total of 544 *Oryza* accessions were used for the genotyping analysis with the C7AIR (S1 Table). The 378 samples contributed by Texas A&M were obtained from the USDA-ARS National Small Grains Collection (Aberdeen, Idaho), the Genetic Stocks-Oryza (GSOR) collection located at the USDA-ARS Dale Bumpers National Rice Research Center (USDA-ARS DBNRRC; Stuttgart, AR), as well as inbred rice breeding materials contributed by the Texas A&M AgriLife Research Center in Beaumont, Texas. The accessions partially overlapped the USDA Rice Mini-Core and Core Subsets [8, 9].This material had representative samples from all 5 subgroups of *O*. *sativa* and *O*. *glaberrima* samples. IRRI’s material contained 23 F1 samples and 48 inbred accessions, and 95 inbred accessions were contributed by the McCouch Lab at Cornell University. Leaf tissue was collected approximately 30–55 days after planting. Samples genotyped at IRRI were processed in the Genotyping Services Lab at IRRI, while samples from other groups were lyophilized and sent to Eurofins Diagnostics, Inc. (www.eurofinsgenomics.eu/en/genotyping-gene-expression/service-platforms/illumina-array-platforms/) for DNA extraction and genotyping.

### Design of the C7AIR

The C7AIR is a beadpool manufactured in liquid phase and stabilized on chips that are commercially available as Illumina Infinium arrays. It was developed by the “International RiceLD Consortium”, a group of investigators from Cornell University, the International Rice Research Institute (IRRI), Texas A&M University, Louisiana State University (LSU), The Dale Bumpers National Rice Research Center (DBNRRC) in Arkansas, the University of Arkansas, and the University of York in the UK.

The Cornell_7K_Array_Infinium_Rice (C7AIR) design represents an improved version of the Cornell_6K_Array_Infinium_Rice (C6AIR) [3]. SNPs that performed poorly on the C6AIR were eliminated, and new SNPs were added specifically to increase the information-content for elite *tropical japonica* breeding material. The selection of SNPs for the C7AIR was based on the following metrics using diversity information from prior work in the McCouch lab: no variants within 10 bp of a target SNP locus; no SNPs within 35 bp of a target SNP locus having a minor homozygote count > 4; no INDELS or repetitive sequences in the target region. The C7AIR included 4,007 SNPs from the C6AIR, 2,056 SNPs from the High Density Rice Array (HDRA) [6], 910 SNPs from the 384-SNP GoldenGate sets [7], 189 SNPs from the 44K array [5] selected to have high information content in U.S. *tropical japonica* rice varieties, and 8 SNPs in genes of interest (S2 Table). Although 7,182 SNPs were submitted to Illumina, this number decreased to 7,098 as some SNPs did not meet their initial quality metrics. Eight gene based SNPs associated with four genes were included in the design of the array: three SNPs for gelatinization temperature in the *starch synthase IIa* or *alk* gene [10, 11], three for apparent amylose content associated with various alleles for the *Wx* gene [12, 13], one for grain length in *Gs3* [14], and one for blast resistance in *Pi-ta* [15].

### Genotyping and SNP allele calling

Genotyping was performed following the manufacturer’s protocol for amplification of DNA, hybridizing to the Infinium II BeadChips, staining with fluorescent dye and scanning to measure the fluorescence intensity of the beadchip. Raw intensity values were sent to each collaborating rice lab and converted to SNP data using Illumina’s GenomeStudio software. The 551 varieties genotyped were then filtered down to 448 based on a call rate above 0.939 (less than 6.1% missing data per sample) and a P10 GC score above 0.45. P10 GC is a score developed by Illumina to identify samples which may have failed genotyping as described in the user manual for GenomeStudio. Upon compiling all data in GenomeStudio, SNPs were manually re-clustered in order to correctly sort the clusters as the correct genotype.

A cluster file for the C7AIR was created using 544 samples (23 F1 samples from IRRI, 48 inbred accessions from IRRI, 95 inbred accessions from Cornell, and 378 inbred accessions from Texas A&M) (S1 Table). These samples were filtered using a p10GC of 0.45 and call rate of 0.939. This removed a total of 63 samples, 50 *O*. *glaberrima*, 12 *O*. *sativa*, and 1 *O*. *meridionalis*. After filtering, the sample clusters were shifted to match three potential clusters (two homozygous and a heterozygous class) or two clusters, when heterozygotes were missing. SNPs were then classified as very high (100% call rate), high (> 400 samples called), poor (<400 samples called), and fail quality (S3 Table). Failed SNPs had less than 10% of samples called, had more than 4 clusters, were completely skewed to one side, or only had one cluster. The genotypic information for each sample consisting of the matrix of 7,098 SNPs across 544 Oryza accessions is available in S4 Table.

### Tree construction and data analysis

Reclustered data was exported from GenomeStudio and imported into TASSEL GUI 5.2.43 where CenteredIBS kinship using a maximum of 6 alleles was calculated [16]. Kinship values were then imported into MEGA7 to create a phylogenetic tree [17]. TASSEL was also used to calculate linkage disequilibrium using a sliding window of 2000 SNPs [16]. Genotyping information was imported to GAPIT where marker density, VanRaeden kinship, and other linkage disequilibrium statistics were determined [18].

A custom R script was used to estimate the number of polymorphic markers between pairwise combinations of lines genotyped with the C7AIR. Genome-wide SNP data from pairs of samples were compared for each individual locus and heterozygosity was calculated. Population subgroups were determined based on the fastStructure output included in S1 Table and filtered to ensure the corresponding principal component was larger than 0.8 for each individual.

### Genome Wide Association Study (GWAS)

A total of 189 varieties of *O*. *sativa* were grown in Beaumont, TX during summer 2017. These varieties were grown using standard agronomic practices in two replications of three-row plots. Five plants of each accession were randomly chosen from the middle row of each plot avoiding the border plants. The height (cm) of each selected rice plant was measured from the base to the neck of the panicle. The heritability of the trait was calculated as: H^2^ = *σ* ^*2*^*g /* (*σ* ^*2*^*g* + *σ* ^*2*^*e/r)*. Homogeneity of variance across the two replications was estimated using Levene’s test [19]. These accessions were genotyped as a subset of the 384 varieties genotyped by Texas A&M.

The GWAS analysis was performed using Mixed Linear Model (MLM) of GAPIT [18]. Both kinship (K) matrix and population structure matrix (Q) were used in the MLM model to account for the relatedness among the genotypes and to reduce false positives. The kinship (K) matrix represented the variance-covariance matrix between the individuals complemented with population structure (Q matrix). The structure data was obtained from the FastSTRUCTURE software [20] and the kinship relationship matrix (K) was obtained from the TASSEL 4.0 software [16].

A significant QTL was defined as a single or a cluster of SNP markers that passed the significance threshold of p <0.001. In general, the extent of LD in rice on average ranges from 100 to 500 kb [21–23]. Based on this assumption, we defined two or more SNPs located within 250 kb as a single QTL. Likewise, gene(s) that are located within 250 kb were considred potentially colocalized with the QTL. Candidate genes were identified using the Q-TARO (QTL Annotation Rice Online) database [24].

## Results and discussion

### Design of the C7AIR

**Fig 1:**
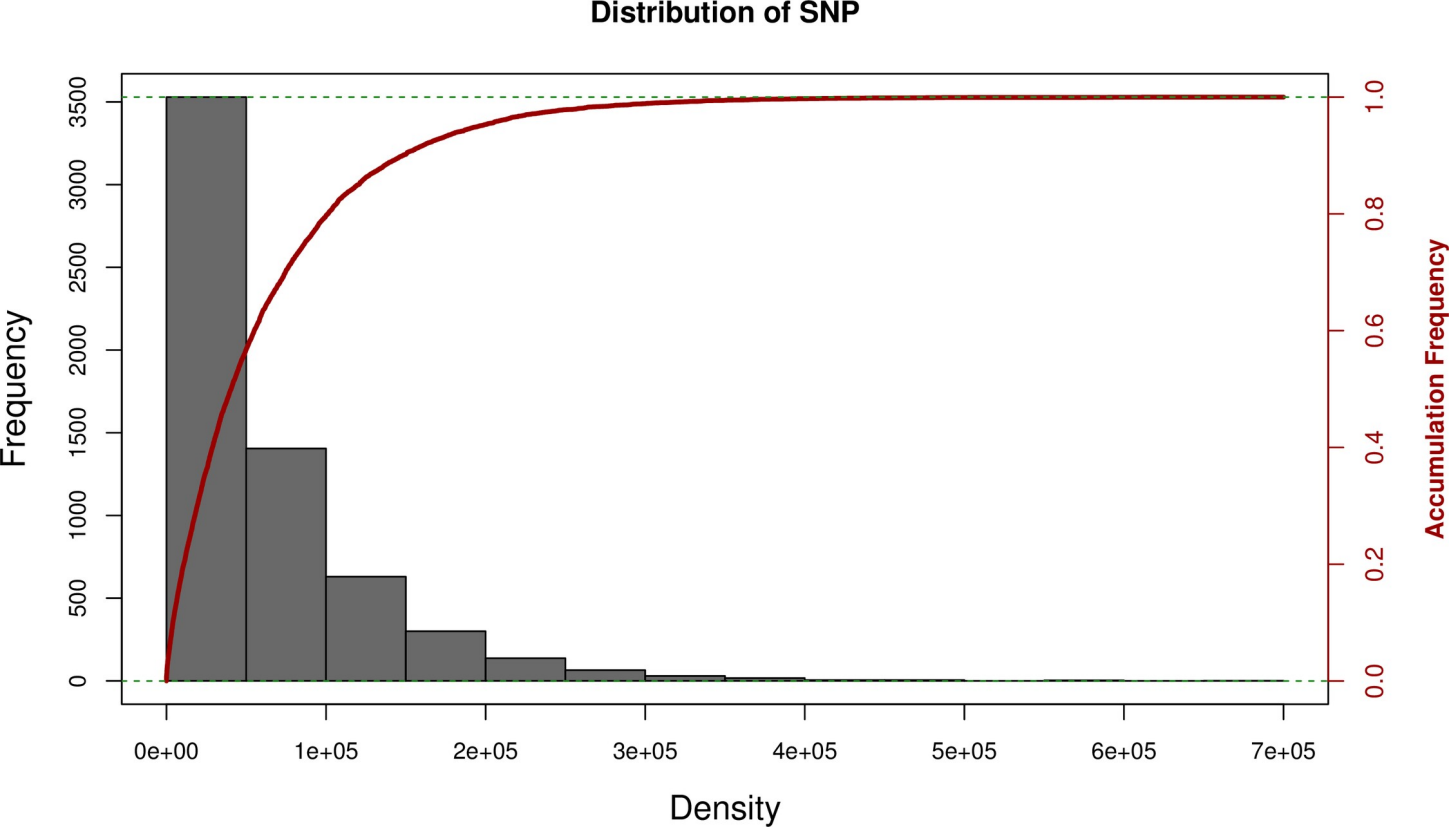
Distribution of gap size between a SNP and its neighbors. The majority of SNPs are within 100 kb of their neighbor, as seen by the histogram of SNP markers on the y-axis and density in basepairs on the x-axis.

The Cornell-IR LD Rice Array (C7AIR) design represents an improved version of the Cornell_6K_Array_Infinium_Rice (C6AIR) [3], and contains a total of 7,098 SNPs. The design of the C7AIR incorporates 4,007 high-quality SNPs from the C6AIR, 2,056 SNPs from the High Density Rice Array (HDRA) [6], 910 SNPs from the 384-SNP GoldenGate sets [7], 189 SNPs from the 44K array [5] selected to have high information content across U.S. *tropical japonica* rice varieties, and 8 SNPs in genes of interest (S2 Table). A summary file containing information about each SNP’s locus_ID, position on the Nipponbare reference genome (IRGSPv1.0), strand, probe sequence (n = 50 bp), flanking sequence (n = 60 bp on either side of the SNP), and source is available as S3 Table. The average SNP density is one SNP per 52 kb, and >50% of SNPs are less than 50 kb away from each other (Fig 1). Subsequent analyses were performed on C7AIR genotype data obtained on a diverse set of 551 rice samples.

### Filtering and manual re-clustering of SNPs

**Fig 2:**
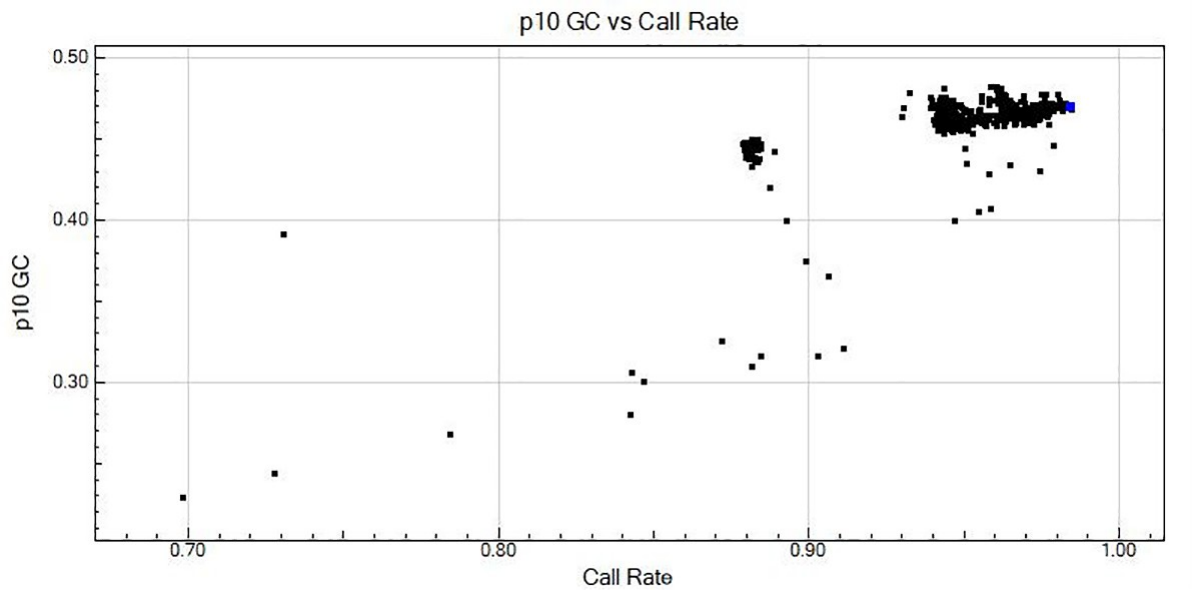
p10 GC versus call rate for all samples. The Illumina p10GC quality metric is shown on the y-axis and the SNP call rate on the x-axis; most samples had p10 GC values >0.45 and call rates > 0.94.

To obtain a sub-set of SNPs with optimal performance for genetic analysis in Asian cultivated rice (*O*. *sativa)*, we masked accessions with low call rates and low quality values, as measured by p10 GC, an Illumina quality parameter (Fig 2). A total of 63 accessions were masked, consisting of 50 *O*. *glaberrima*, 12 *O*. *sativa*, and one *O*. *meridionalis* (wild accession). Low call rates on SNP arrays are typically due to sequence variation in the regions flanking the target SNPs. It is noteworthy that all *O*. *rufipogon* and *O*. *nivara* samples in this study had high call rates (> 0.94), similar to *O*. *sativa*, and 0.45 p10 GC, while *O*. *glaberrima* accessions had lower call rates (~0.88), consistent with the greater evolutionary distance between *O*. *sativa* and *O*. *glaberrima* as reflected in the kinship matrix (S5 Table).

After masking poor-performing DNA samples, the SNPs were filtered based on quality parameters. Most of the SNPs (n = 6514) passed initial quality control, but the number of heterozygous calls for each locus was very low due to the highly homozygous nature of rice as an inbred crop. This is challenging when developing an automated cluster file with the Illumina GenomeStudio software because the algorithm often cannot correctly identify three clusters when there are few heterozgyotes. Therefore, GenomeStudio clusters were manually corrected and saved as a new cluster file, as described below in the Methods section. SNPs filtered out using this process may still be beneficial, especially for diversity analysis within wild species.

### Diversity analysis

**Fig 3:**
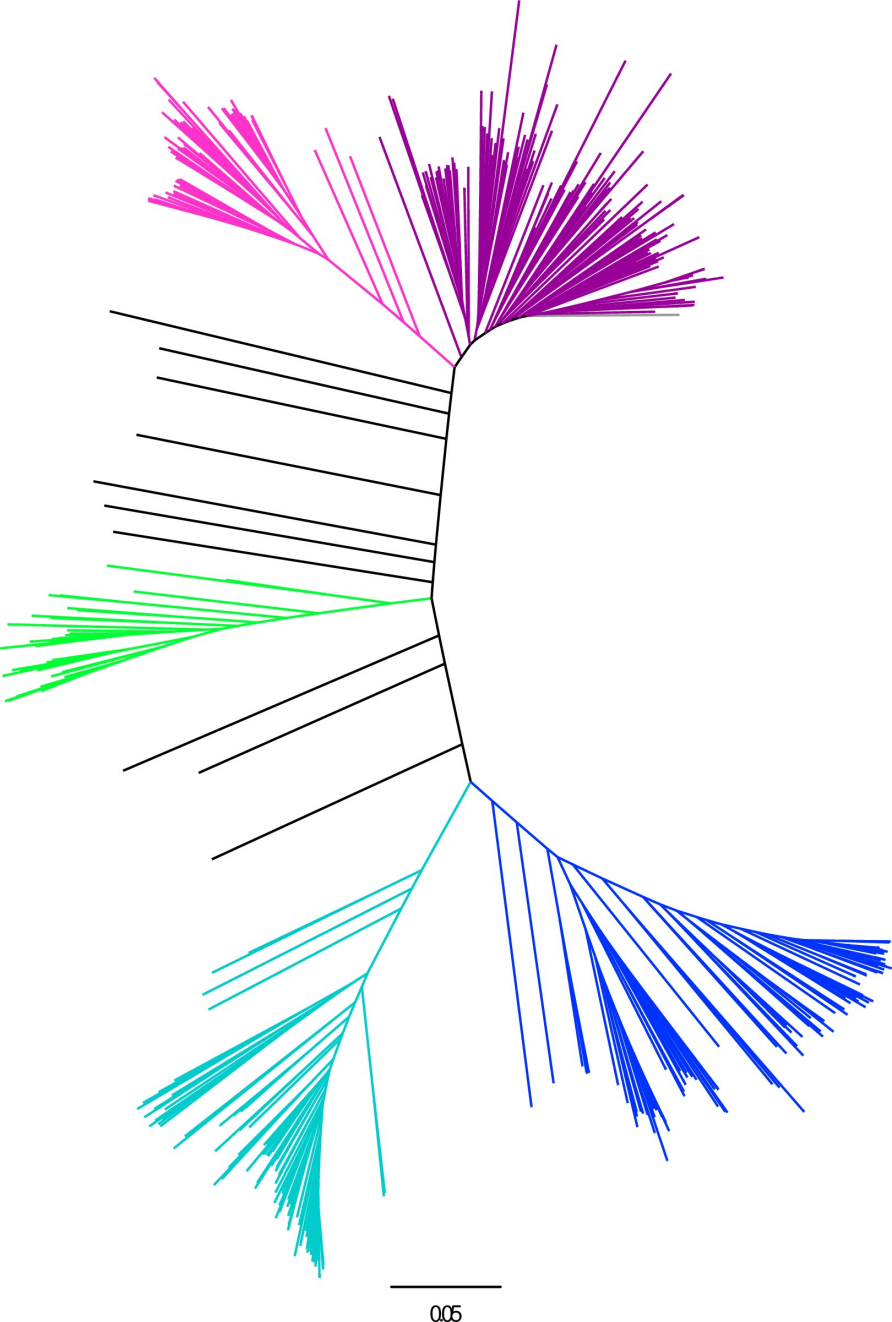
Phylogenetic tree from 6,514 SNPs across 551 *Oryza sativa* accessions. The five subgroups of *O*. *sativa* are shown: *indica*: dark purple, *aus*: light purple, *aromatic*: green, *temperate japonica*: turquoise, *tropical japonica*: blue, admixed: black.

**Fig 4:**
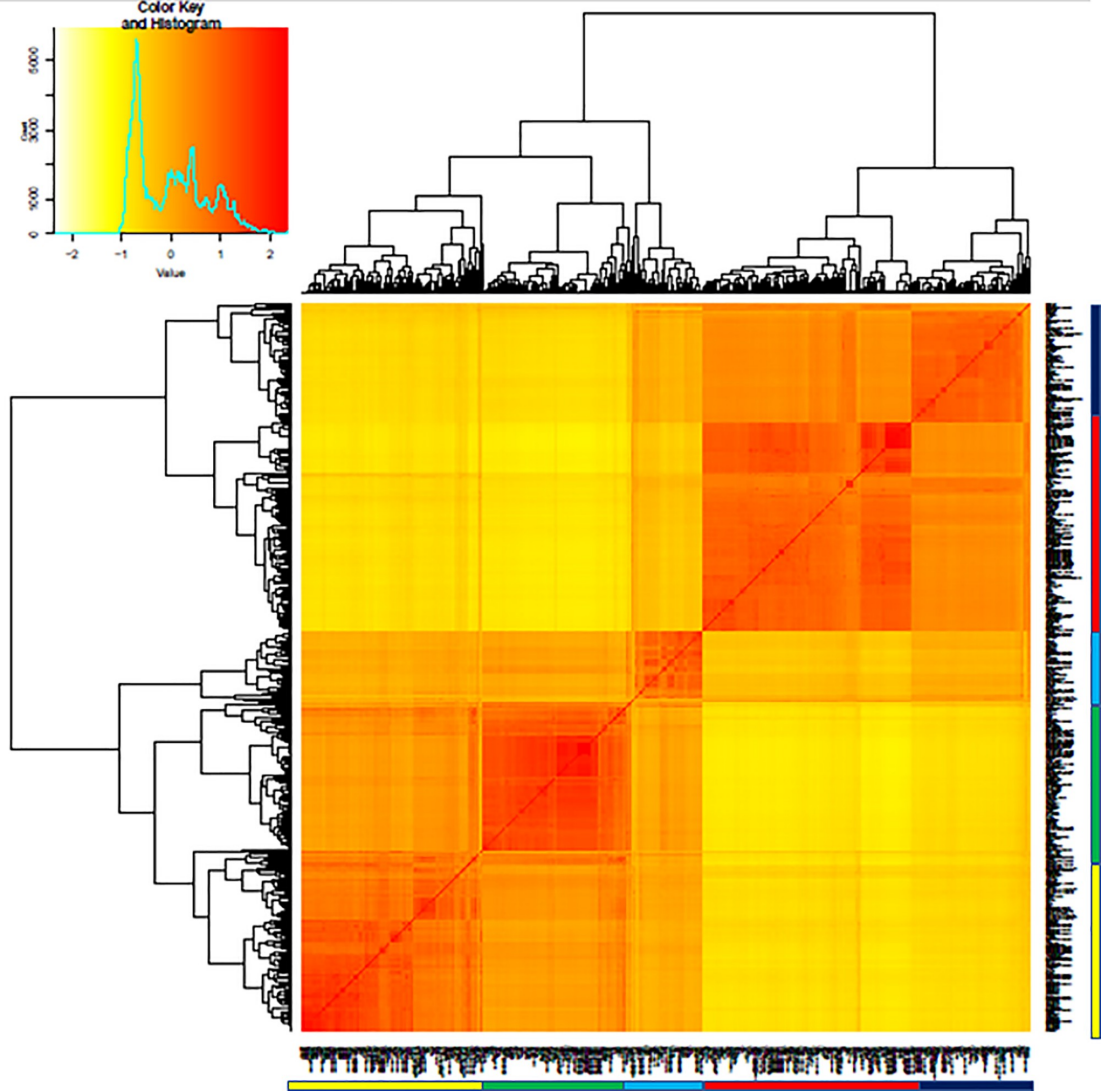
VanRaeden kinship heat map. The relatedness of individuals compared to each other is shown, with the legend identifying subgroups of *O*. *sativa*.

The C7AIR successfully differentiated the 5 major subgroups of *O*. *sativa* using Centered IBS (Fig 3) and VanRaeden kinship measurements (Fig 4). The Southern U.S. varieties appropriately clustered with the *tropical japonica* subpopulation, as expected based on their breeding history. When pairwise comparisons were performed between *O*.*sativa* and *O*. *glaberrima*, *O*.*sativa* was found to have the highest nucleotide diversity. The C7AIR was best able to differentiate individuals within the *indica* subgroup with informativeness decreasing with *tropical japonica*, *aus*, *temperate japonica* and least informative for *aromatic*. This SNP array readily differentiates *temperate japonica* from *indica*, while the fewer polymorphisms were observed between *indica* and *aus* (S6 Table).

### Genome wide association study

Genome wide association studies (GWAS) take advantage of historical LD in a collection of diverse lines in an effort to correlate regions of the genome with specific phenotypes [21]. The average extent of LD in *O*. *sativa* ranges from approximately 100 kb in *aus* and *indica*, to 500 kb in *tropical japonica* and *temperate japonica* [23, 25]. USDA scientists have created core and mini-core collections of rice germplasm for use in association mapping studies [8, 9]. To test the utility of the C7AIR for GWAS, the array was used to genotype a set of 189 lines, representing a subset of the USDA core and mini core collections along with additional accessions from the the USDA National Small Grains Collection (NSGC), and the lines were also phenotyped for plant height.

The plant height data showed a wide distribution ranging from 32.9 to 150.35 cm with a population mean of 103.82 cm and standard deviation of 28.78 cm. The heritability was estimated to be 97.4; which showed that the trait was mostly genetically controlled. This was also further confirmed by the the Levene’s test result which shows that the variances across replications were insignificant (p-value = 0.1375); consequently the plant height data were averaged across replications and used as representative phenotypic data for the GWAS study. A total of six subpopulations were obtained from FastStructure. This population structure and the kinship matrix obtained were used in the plant height GWAS.

**Fig 5:**
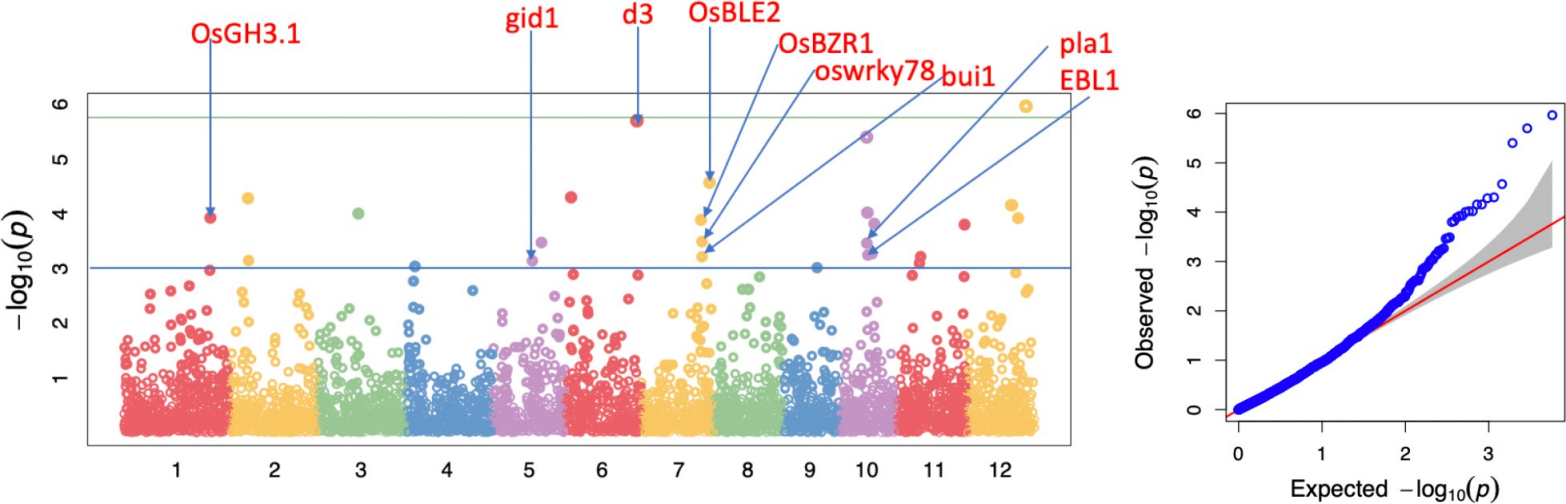
GWAS results for plant height using SNPs from the C7AIR. The manhattan plot shows the level of significance for SNPs correlated with plant height from the GWAS analysis (numbers across the x-axis indicate the chromosome). The Q-Q plot is shown at right. The blue line indicates the p <0.001 threshold and the green line indicates the Bonferroni correction threshold.

A total of 20 QTLs were detected using the p-value of 0.001 significant threshold; among them 14 were detected with a FDR < 0.1 and 4 were detected with FDR<0.05 (Table 1; Fig 5). The QTL were defined based on a single significant SNP or a cluster of significant SNPs within 250 kb, with the exception of the QTL on chromosome 10, *qPHT-10-1*. This QTL was identified by a clustered of 5 SNPs having distances of less than 250 kb between each other, within a total region of 485.32 kb. Out of 20 QTLs, 8 of them were found in the vicinity of genes previously reported to be associated with plant height or dwarfism; while the rest of the QTLs are potentially novel, including the QTL on chromosome 12 (*qPHT-12-3*) that has the most significant SNP in our study. Six of these genes were located less than 250 kb to the nearest significant SNPs, while the gene *pla1* was 269.8 kb and the gene *OsGH3*.*1* was located 300.5 kb from its nearest significant SNP. Interstingly, two of the genes were detected within distances less than 15 kb to the nearest SNP peaks (as described below).

**Table 1:** QTLs for plant height detected by GWAS and potentially colocalized genes.

QTL ID	Chr.	Position	p-value	FDR	R^2^	Gene	Start	End	Function	Reference
*qPHT-1*	1	35367274	0.00012	0.0534148	5.16	*OsGH3*.*1* (Os01g0785400)	35064294	35066779	Auxin content, cell elongation, resistance to *Magnaporthe grisea* infection	Dominggo et al. 2009
*qPHT-2*	2	7487524	5.22E-05	0.0502478	5.73					
	2	7699096	0.00072	0.1674799	3.95					
*qPHT-3*	3	16688962	9.92E-05	0.0520817	5.28					
*qPHT-4*	4	3411480	0.00092	0.1899119	3.79					
	4	3419276	0.00091	0.1899119	3.79					
*qPHT-5-1*	5	16354910	0.00073	0.1674799	3.94					
*qPHT-5-2*	5	20055547	0.00034	0.1051188	4.46	*gid1* (Os05g0407500)	19875298	19878096	Dwarfism, Gibberellin sensitivity	Ueguchi-Tanaka et al. 2005
*qPHT-6-1*	6	2553144	5.01E-05	0.0502478	5.76	*d3* (Os06g0154200)	2779789	2784227	Tillering dwarf, tiller bud outgrowth	Ishikawa et al. 2005
*qPHT-6-2*	6	29397359	2.00E-06	0.00578	8.05					
*qPHT-7-1*	7	24304110	0.00013	0.0534303	5.10	*OsBZR1* (Os07g0580500); *oswrky78* (Os07g0583700)	24144128; 24314295	24145465; 24319767	Dwarfism, leaf angle, Brassinosteroid sensitivity; Dwarfism, grain size, cell elongation	Bai et al. 2007; Zhang et al. 2011
*qPHT-7-2*	7	24749346	0.00032	0.1051188	4.48	*bui1* (Os07g0596300)	24926636	24933527	Cell division and expansion, actin organization	Yang et al. 2011
	7	24759254	0.00061	0.1535709	4.06					
*qPHT-7-3*	7	27797259	2.70E-05	0.0390099	6.19	*OsBLE2* (Os07g0650600)	27857872	27861552	Dwarfism, leaf angle, Brassinosteroid sensitivity	Yang et al. 2003
*qPHT-9*	9	13656075	0.00097	0.1922282	3.75					
*qPHT-10-1*	10	11173303	3.97E-06	0.007642	7.55					
	10	11251070	0.00035	0.1051188	4.44					
	10	11372537	9.56E-05	0.0520817	5.31					
	10	11600834	9.56E-05	0.0520817	5.31					
	10	11658623	0.00057	0.1535709	4.11					
*qPHT-10-2*	10	13335008	0.00054	0.1535709	4.14	*EBL1* (Os10g0390800)	13348115	13349483	Internode elongation	Iwamoto and Takano 2010
*qPHT-10-3*	10	14287709	0.00015	0.0570201	4.99	*pla1* (Os10g0403000)	14016190	14017943	Plastochron and phyllotaxy, dwarfism	Miyoshi et al. 2004
*qPHT-11-1*	11	9607864	0.00081	0.1787786	3.87					
	11	10116476	0.00061	0.1535709	4.06					
*qPHT-11-2*	11	27998933	0.00016	0.0570201	4.97					
*qPHT-12-1*	12	17958383	6.98E-05	0.0504057	5.53					
	12	18353718	6.98E-05	0.0504057	5.53					
*qPHT-12-2*	12	20843326	0.00012	0.0534148	5.15					
*qPHT-12-3*	12	24043382	1.08E-06	0.00578	8.49					

A QTL was detected on chromosome 1 at position 35.367 Mb (*qPHT-1*) which is in the vicinity of *OsGH3*.*1* (35.067Mb). Overexpression of the *OsGH3*.*1* gene in rice causes dwarfism and decreased free auxin content and cell elongation [26]. A QTL on chromosome 5 at position 20.056 Mb, *qPHT-5-2*, potentially colocalized with *gid1* (19.878 Mb), which encodes for an unknown protein similar to lipases and may act as a receptor mediating GA signaling in rice which leads to dwarfism [27]. Another QTL on chromosome 6 at position 2.553 Mb, *qPHT-6-1*, was located nearby the *d3* gene, which causes tillering dwarf mutants and an increase in tiller number [28]. There were three QTLs detected on chromosome 7, and all of them potentially colocalized with known genes. The first QTL at position 24.304 Mb, *qPHT-7-1*, was detected only 10.2 kb away from *oswrky78* (24.320 Mb)—a gene that may regulate stem elongation and seed development [29]. This QTL was also detected at 158.7 kb away from *OsBZR1* (24.145 Mb), and supression of this gene leads to dwarfism and alters brassinosteroids (BR) responses in rice [30]. The two closely linked-genes within the region of the QTL may potentially contribute individually or together to the QTL phenotype. The second QTL at significant SNP peaks positioned at 24.749 Mb and 24.759 Mb, *qPHT-7-2*, was in the vicinity of *bui1* (*FH5/BENT UPPERMOST INTERNODE1*; 24.934 Mb). This gene encodes a formin-type actin nucleation factor and affects cell expansion and plant morphogenesis in rice, including bent uppermost internode, dwarfism, wavy panicle rachis, and increased gravitropic response [31]. The third QTL at position 27.797 Mb, *qPHT-3-1*, was only 60.6 kb away from *OsBLE2* (27.858 Mb). This gene may be involved in brassinolide (BL)-regulated growth and development processes in rice. Transgenic rice expressiong antisense of this gene demonstrates repressed growth [32]. Another QTL on chromosome 10 at position 13.335 Mb, *qPHT10-2*, was detected only 13.1 kb away from the *EBL1* gene (13.348 Mb), and silencing of this gene leads to plants with reduced plant height [33]. Lastly, another QTL on chrosome 10 at position 14.288 Mb, *qPHT-10-3*, was in the proximity of the *plastochron (PLA)1* gene (14.018 Mb). *PLA1* was isolated by map-based cloning and encodes a cytochrome P450, CYP78A11, which potentially catalyzes substances controlling plant development, including leaf primordia, bracts of the panicle, and elongating internodes [34]. Although the causal relationships of these genes with the significant SNP markers will need further investigation to confirm, these findings indicate that the C7AIR is useful for GWAS, and that it can identify key regions of interest for futher study. While the relatively low density of this array may decrease resolution for locating causal genes underlying the GWAS-QTLs, the high extent of LD in rice populations (100-500kb) is often the more limiting factor.

### Implementation of the C7AIR

The C7AIR is available from Illumina for rice genetics research and breeding applications around the world. Since its release it has been used to develop inter-specific chromosome segment substitution lines (CSSLs) involving crosses between *O*. *sativa* and its wild relatives, fingerprint diverse accessions from genebanks, and for QTL and association mapping. Although cost comparisons can be difficult to make between genotyping platforms due to differences in SNP call rates, informativeness, and varying prices depending on sample commitments, the C7AIR gives the global research community a uniform tool to easily compare across research programs while being of high enough density to meet the requirements for a variety of research applications. It has also served as a resource to identify informative SNPs that can be deployed in low-cost, low-density genotyping assays such as KASP assays widely used for marker-assisted selection in forward breeding, or the 1K-Rice Custom Amplicon (1k-RiCA) SNP assay (https://gsl.irri.org/services/genotyping/rica) used for genomic selection [35].

## Conclusions

The C7AIR was designed at Cornell University and established as an Illumina consortium array by public sector researchers and breeders to meet their collective needs for an array that would provide reliable, rapid and efficient genotyping data for rice. The C7AIR has since been used to successfully genotype >10,000 samples of rice across the consortium partners. The C7AIR is designed to be informative for detecting genome-wide polymorphism among individuals within the *indica*, *aus*, and *tropical japonica* subpopulations of *O*. *sativa*, between pairwise combinations of *indica*, *aus*, *tropical japonica*, *temperate japonica*, or *aromatic* (Group V) subpopulations, and between *O*. *sativa* and *O*. *rufipogon*. The array is less informative for detecting polymorphism within *temperate japonica* or the *aromatic* (Group V) subpopulation, and was not designed to target SNPs specific to *O*. *glaberrima* (S6 Table). While all fixed arrays have inherent bias due to the design priorities used to select SNPs, the C7AIR provides a useful genotyping tool for many research and breeding applications in *O*. *sativa*, and is widely used by the global rice community for genetic mapping, DNA fingerprinting, development of genetic stocks, marker assisted selection and genomic selection. It is also an excellent source of critical information about SNP reliability and variation in rice that can be used to select SNPs for conversion to new genotyping platforms.

## Supporting information

S1 Table Sample names and inferred ancestry/subpopulation assignments from fastStructure analysis for 544 *Oryza* accessions run with the C7AIR. (XLSX)

S2 Table SNP IDs, target genes, and target traits for 8 trait-specific SNP markers included in the C7AIR. (XLSX)

S3 Table The SNP manifest file, with SNP ID, quality, chromosomal positions, probe and flanking sequence information for 7,098 SNP loci on the C7AIR. (XLSX)

S4 Table SNP genotype data matrix for 7,098 SNPs across 544 *Oryza* accessions, along with plant height data for a subset of accessions. (XLSX)

S5 Table Kinship values between the accessions generated using the highest performing SNPs. (XLSX)

S6 Table The average and maximum number of polymorphisms between and within *Oryza* species and between and within subgroups of *O*. *sativa*. (XLSX)
